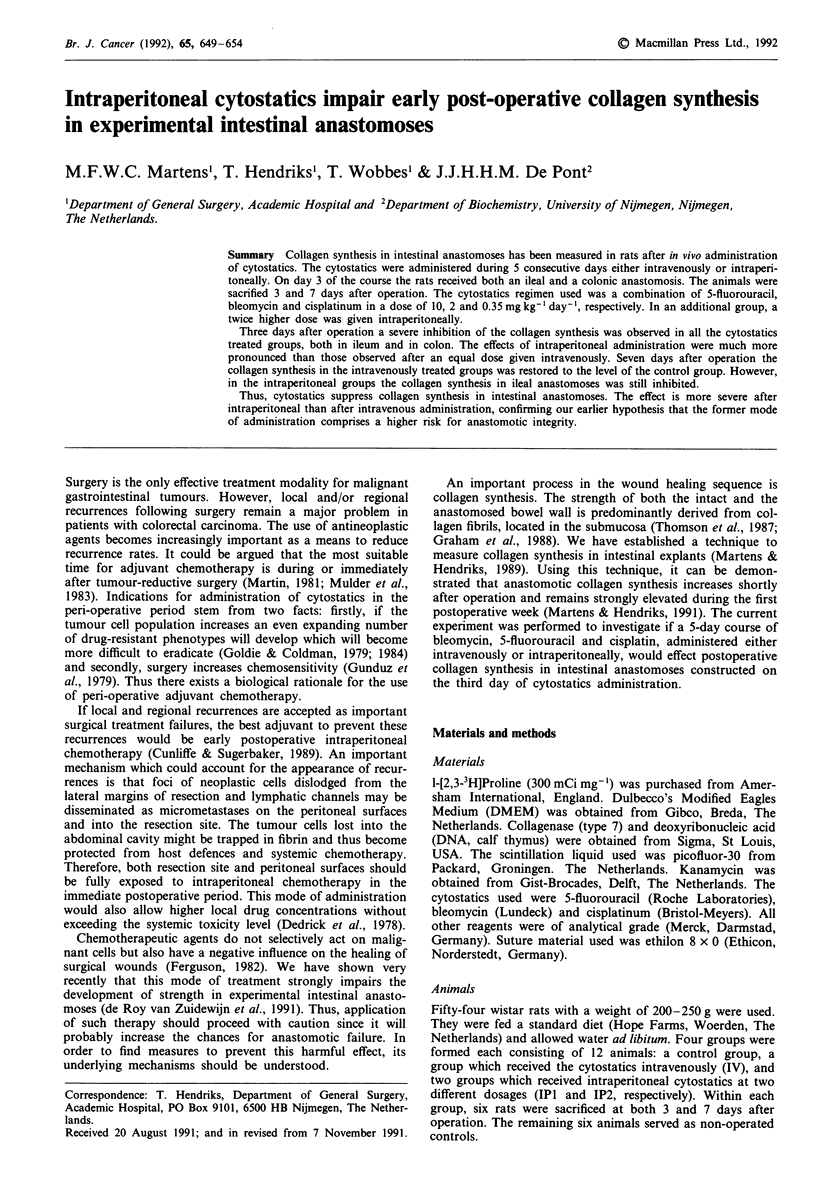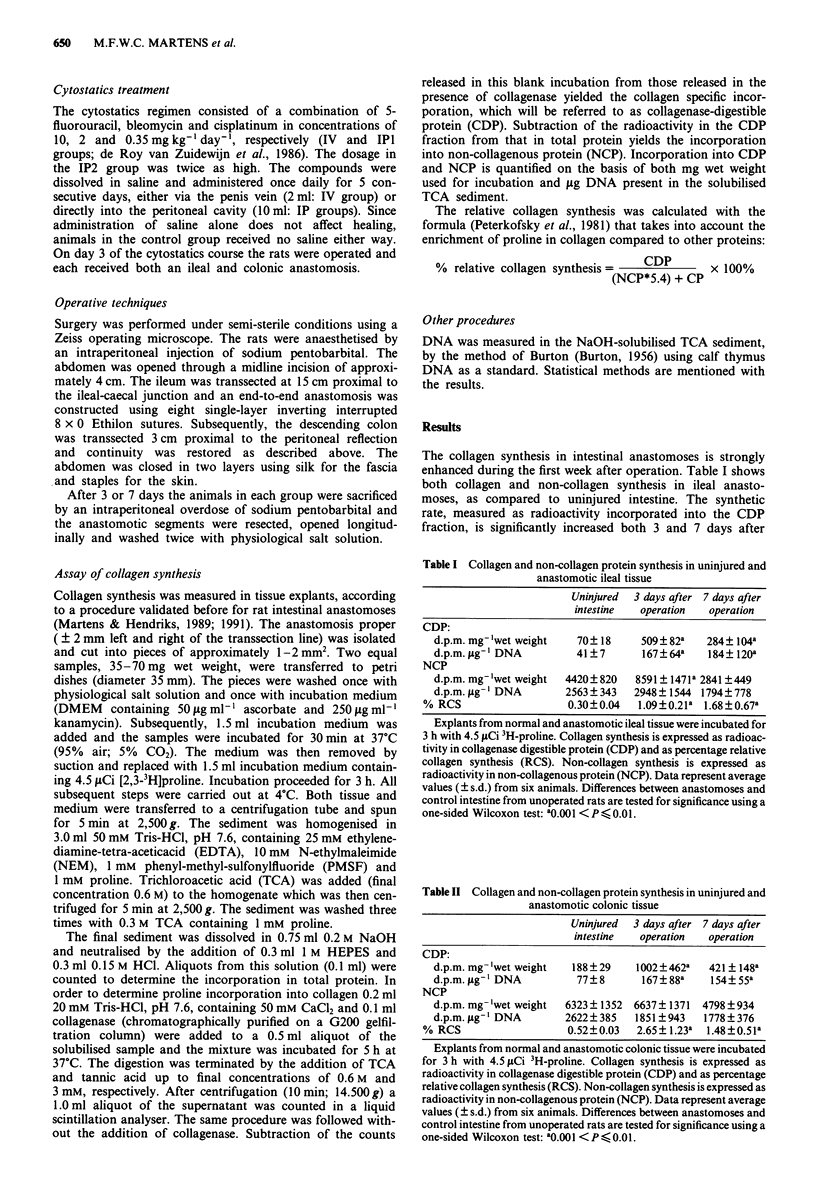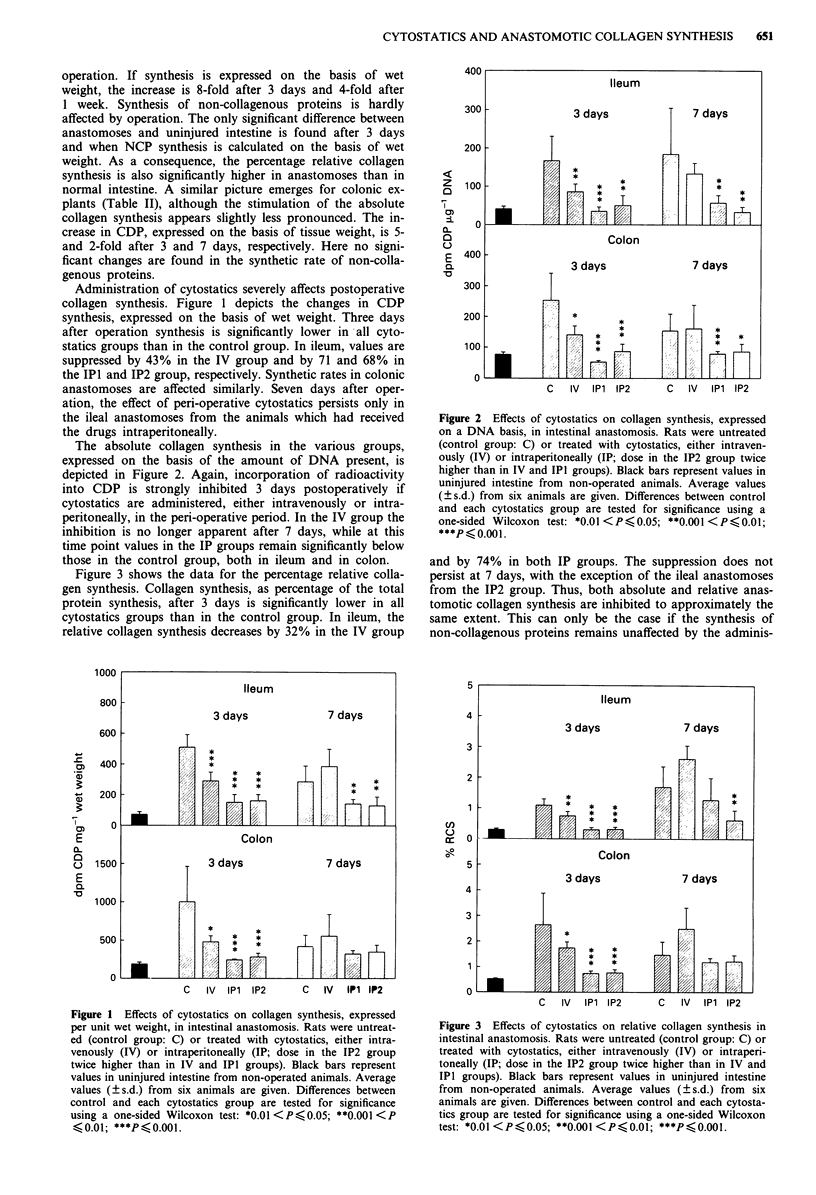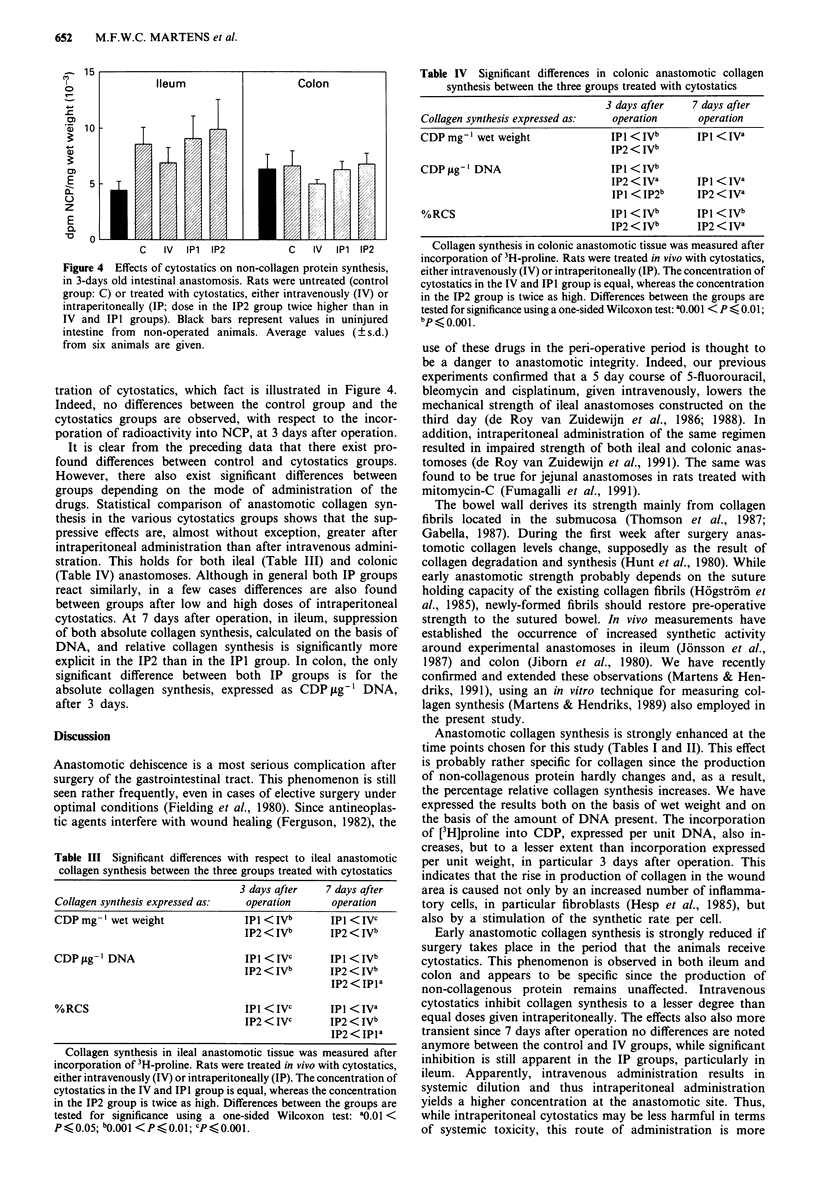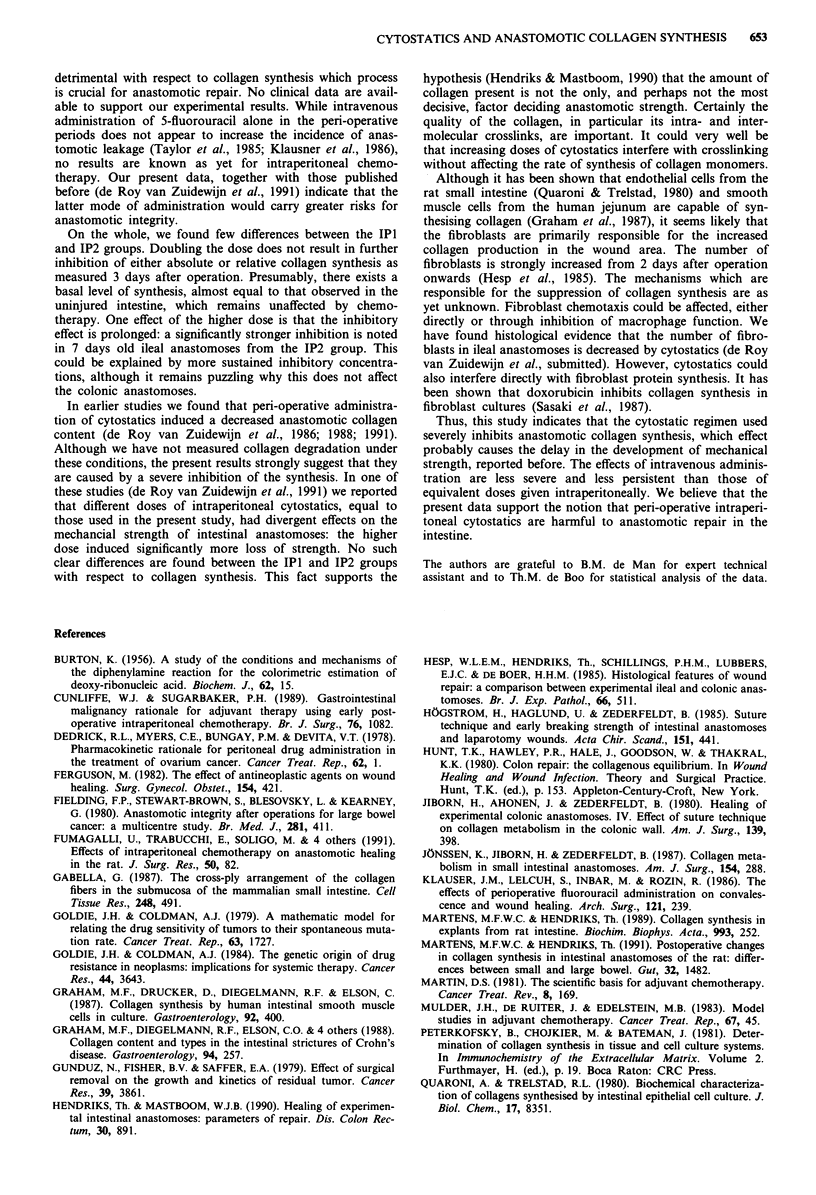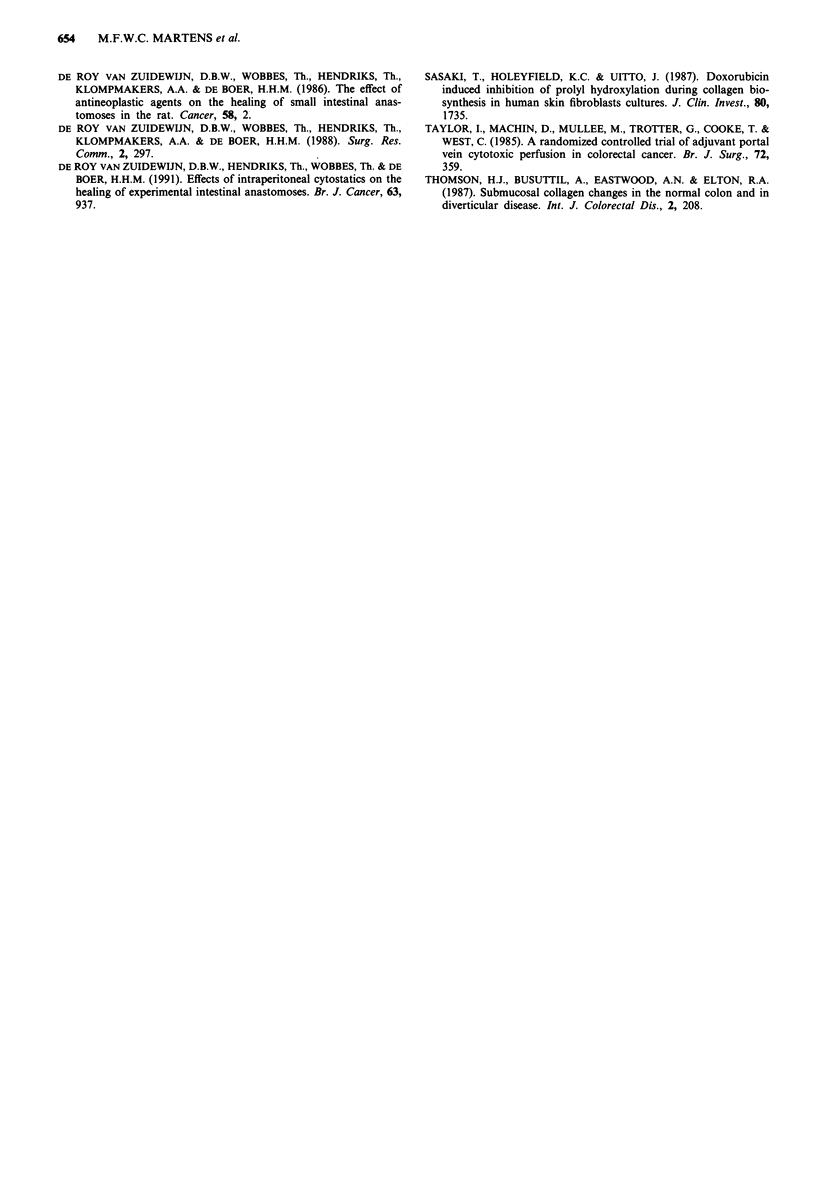# Intraperitoneal cytostatics impair early post-operative collagen synthesis in experimental intestinal anastomosesP6.

**DOI:** 10.1038/bjc.1992.139

**Published:** 1992-05

**Authors:** M. F. Martens, T. Hendriks, T. Wobbes, J. J. De Pont

**Affiliations:** Department of General Surgery, Academic Hospital, Nijmegen, The Netherlands.

## Abstract

Collagen synthesis in intestinal anastomoses has been measured in rats after in vivo administration of cytostatics. The cytostatics were administered during 5 consecutive days either intravenously or intraperitoneally. On day 3 of the course the rats received both an ileal and a colonic anastomosis. The animals were sacrificed 3 and 7 days after operation. The cytostatics regimen used was a combination of 5-fluorouracil, bleomycin and cisplatinum in a dose of 10, 2 and 0.35 mg kg-1 day-1, respectively. In an additional group, a twice higher dose was given intraperitoneally. Three days after operation a severe inhibition of the collagen synthesis was observed in all the cytostatics treated groups, both in ileum and in colon. The effects of intraperitoneal administration were much more pronounced than those observed after an equal dose given intravenously. Seven days after operation the collagen synthesis in the intravenously treated groups was restored to the level of the control group. However, in the intraperitoneal groups the collagen synthesis in ileal anastomoses was still inhibited. Thus, cytostatics suppress collagen synthesis in intestinal anastomoses. The effect is more severe after intraperitoneal than after intravenous administration, confirming our earlier hypothesis that the former mode of administration comprises a higher risk for anastomotic integrity.


Br. J. Cancer (1992), 65, 649–654 © Macmillan Press Ltd., 1992

### Intraperitoneal cytostatics impair early post-operative collagen synthesis in experimental intestinal anastomoses

M.F.W.C. Martens[1], T. Hendriks[1], T. Wobbes[1] & J.J.H.H.M. De Pont[2]

[1]*Department of General Surgery, Academic Hospital and* [2]*Department of Biochemistry, University of Nijmegen, Nijmegen, The Netherlands.*

**Summary** Collagen synthesis in intestinal anastomoses has been measured in rats after *in vivo* administration of cytostatics. The cytostatics were administered during 5 consecutive days either intravenously or intraperitoneally. On day 3 of the course the rats received both an ileal and a colonic anastomosis. The animals were sacrified 3 and 7 days after operation. The cytostatics regimen used was a combination of 5-fluorouracil, bleomycin and cisplatinum in a dose of 10, 2 and 0.35 mg $kg^{-1}$ $day^{-1}$, respectively. In an additional group, a twice higher dose was given intraperitoneally.

Three days after operation a severe inhibition of the collagen synthesis was observed in all the cytostatics treated groups, both in ileum and in colon. The effects of intraperitoneal administration were much more pronounced than those observed after an equal dose given intravenously. Seven days after operation the collagen synthesis in the intravenously treated groups was restored to the level of the control group. However, in the intraperitoneal groups the collagen synthesis in ileal anastomoses was still inhibited.

Thus, cytostatics suppress collagen synthesis in intestinal anastomoses. The effect is more severe after intraperitoneal than after intravenous administration, confirming our earlier hypothesis that the former mode of administration comprises a higher risk for anastomotic integrity.

Surgery is the only effective treatment modality for malignant gastrointestinal tumours. However, local and/or regional recurrences following surgery remain a major problem in patients with colorectal carcinoma. The use of antineoplastic agents becomes increasingly important as a means to reduce recurrence rates. It could be argued that the most suitable time for adjuvant chemotherapy is during or immediately after tumour-reductive surgery (Martin, 1981; Mulder *et al.*, 1983). Indications for administration of cytostatics in the peri-operative period stem from two facts: firstly, if the tumour cell population increases an even expanding number of drug-resistant phenotypes will develop which will become more difficult to eradicate (Goldie & Coldman, 1979; 1984) and secondly, surgery increases chemosensitivity (Gunduz *et al.*, 1979). Thus there exists a biological rationale for the use of peri-operative adjuvant chemotherapy.

If local and regional recurrences are accepted as important surgical treatment failures, the best adjuvant to prevent these recurrences would be early postoperative intraperitoneal chemotherapy (Cunliffe & Sugerbaker, 1989). An important mechanism which could account for the appearance of recurrences is that foci of neoplastic cells dislodged from the lateral margins of resection and lymphatic channels may be disseminated as micrometastases on the peritoneal surfaces and into the resection site. The tumour cells lost into the abdominal cavity might be trapped in fibrin and thus become protected from host defences and systemic chemotherapy. Therefore, both resection site and peritoneal surfaces should be fully exposed to intraperitoneal chemotherapy in the immediate postoperative period. This mode of administration would also allow higher local drug concentrations without exceeding the systemic toxicity level (Dedrick *et al.*, 1978).

Chemotherapeutic agents do not selectively act on malignant cells but also have a negative influence on the healing of surgical wounds (Ferguson, 1982). We have shown very recently that this mode of treatment strongly impairs the development of strength in experimental intestinal anastomoses (de Roy van Zuidewijn *et al.*, 1991). Thus, application of such therapy should proceed with caution since it will probably increase the chances for anastomotic failure. In order to find measures to prevent this harmful effect, its underlying mechanisms should be understood.

Correspondence: T. Hendriks, Department of General Surgery, Academic Hospital, PO Box 9101, 6500 HB Nijmegen, The Netherlands.

Received 20 August 1991; and in revised from 7 November 1991.

An important process in the wound healing sequence is collagen synthesis. The strength of both the intact and the anastomosed bowel wall is predominantly derived from collagen fibrils, located in the submucosa (Thomson *et al.*, 1987; Graham *et al.*, 1988). We have established a technique to measure collagen synthesis in intestinal explants (Martens & Hendriks, 1989). Using this technique, it can be demonstrated that anastomotic collagen synthesis increases shortly after operation and remains strongly elevated during the first postoperative week (Martens & Hendriks, 1991). The current experiment was performed to investigate if a 5-day course of bleomycin, 5-fluorouracil and cisplatin, administered either intravenously or intraperitoneally, would effect postoperative collagen synthesis in intestinal anastomoses constructed on the third day of cytostatics administration.

### Materials and methods

### Materials l-[2,3-$^3$H]Proline (300 mCi $mg^{-1}$) was purchased from Amersham International, England. Dulbecco's Modified Eagles Medium (DMEM) was obtained from Gibco, Breda, The Netherlands. Collagenase (type 7) and deoxyribonucleic acid (DNA, calf thymus) were obtained from Sigma, St Louis, USA. The scintillation liquid used was picofluor-30 from Packard, Groningen. The Netherlands. Kanamycin was obtained from Gist-Brocades, Delft, The Netherlands. The cytostatics used were 5-fluorouracil (Roche Laboratories), bleomycin (Lundeck) and cisplatinum (Bristol-Meyers). All other reagents were of analytical grade (Merck, Darmstad, Germany). Suture material used was ethilon 8 × 0 (Ethicon, Norderstedt, Germany).

### Animals

Fifty-four wistar rats with a weight of 200–250 g were used. They were fed a standard diet (Hope Farms, Woerden, The Netherlands) and allowed water *ad libitum*. Four groups were formed each consisting of 12 animals: a control group, a group which received the cytostatics intravenously (IV), and two groups which received intraperitoneal cytostatics at two different dosages (IP1 and IP2, respectively). Within each group, six rats were sacrificed at both 3 and 7 days after operation. The remaining six animals served as non-operated controls.

*Cytostatics treatment*

The cytostatics regimen consisted of a combination of 5-fluorouracil, bleomycin and cisplatinum in concentrations of 10, 2 and 0.35 mg $kg^{-1}$ $day^{-1}$, respectively (IV and IP1 groups; de Roy van Zuidewijn *et al.*, 1986). The dosage in the IP2 group was twice as high. The compounds were dissolved in saline and administered once daily for 5 consecutive days, either via the penis vein (2 ml: IV group) or directly into the peritoneal cavity (10 ml: IP groups). Since administration of saline alone does not affect healing, animals in the control group received no saline either way. On day 3 of the cytostatics course the rats were operated and each received both an ileal and colonic anastomosis.

*Operative techniques*

Surgery was performed under semi-sterile conditions using a Zeiss operating microscope. The rats were anaesthetised by an intraperitoneal injection of sodium pentobarbital. The abdomen was opened through a midline incision of approximately 4 cm. The ileum was transsected at 15 cm proximal to the ileal-caecal junction and an end-to-end anastomosis was constructed using eight single-layer inverting interrupted 8 × 0 Ethilon sutures. Subsequently, the descending colon was transsected 3 cm proximal to the peritoneal reflection and continuity was restored as described above. The abdomen was closed in two layers using silk for the fascia and staples for the skin.

After 3 or 7 days the animals in each group were sacrificed by an intraperitoneal overdose of sodium pentobarbital and the anastomotic segments were resected, opened longitudinally and washed twice with physiological salt solution.

*Assay of collagen synthesis*

Collagen synthesis was measured in tissue explants, according to a procedure validated before for rat intestinal anastomoses (Martens & Hendriks, 1989; 1991). The anastomosis proper (± 2 mm left and right of the transsection line) was isolated and cut into pieces of approximately 1–2 $mm^2$. Two equal samples, 35–70 mg wet weight, were transferred to petri dishes (diameter 35 mm). The pieces were washed once with physiological salt solution and once with incubation medium (DMEM containing 50 μg $ml^{-1}$ ascorbate and 250 μg $ml^{-1}$ kanamycin). Subsequently, 1.5 ml incubation medium was added and the samples were incubated for 30 min at 37°C (95% air; 5% $CO_2$). The medium was then removed by suction and replaced with 1.5 ml incubation medium containing 4.5 μCi [2,3-$^3$H]proline. Incubation proceeded for 3 h. All subsequent steps were carried out at 4°C. Both tissue and medium were transferred to a centrifugation tube and spun for 5 min at 2,500 *g*. The sediment was homogenised in 3.0 ml 50 mM Tris-HCl, pH 7.6, containing 25 mM ethylene-diamine-tetra-aceticacid (EDTA), 10 mM N-ethylmaleimide (NEM), 1 mM phenyl-methyl-sulfonylfluoride (PMSF) and 1 mM proline. Trichloroacetic acid (TCA) was added (final concentration 0.6 M) to the homogenate which was then centrifuged for 5 min at 2,500 *g*. The sediment was washed three times with 0.3 M TCA containing 1 mM proline.

The final sediment was dissolved in 0.75 ml 0.2 M NaOH and neutralised by the addition of 0.3 ml 1 M HEPES and 0.3 ml 0.15 M HCl. Aliquots from this solution (0.1 ml) were counted to determine the incorporation in total protein. In order to determine proline incorporation into collagen 0.2 ml 20 mM Tris-HCl, pH 7.6, containing 50 mM $CaCl_2$ and 0.1 ml collagenase (chromatographically purified on a G200 gelfiltration column) were added to a 0.5 ml aliquot of the solubilised sample and the mixture was incubated for 5 h at 37°C. The digestion was terminated by the addition of TCA and tannic acid up to final concentrations of 0.6 M and 3 mM, respectively. After centrifugation (10 min; 14.500 *g*) a 1.0 ml aliquot of the supernatant was counted in a liquid scintillation analyser. The same procedure was followed without the addition of collagenase. Subtraction of the counts released in this blank incubation from those released in the presence of collagenase yielded the collagen specific incorporation, which will be referred to as collagenase-digestible protein (CDP). Subtraction of the radioactivity in the CDP fraction from that in total protein yields the incorporation into non-collagenous protein (NCP). Incorporation into CDP and NCP is quantified on the basis of both mg wet weight used for incubation and μg DNA present in the solubilised TCA sediment.

The relative collagen synthesis was calculated with the formula (Peterkofsky *et al.*, 1981) that takes into account the enrichment of proline in collagen compared to other proteins:

$$\% \text{ relative collagen synthesis} = \frac{\text{CDP}}{(\text{NCP}*5.4) + \text{CP}} \times 100\%$$

*Other procedures*

DNA was measured in the NaOH-solubilised TCA sediment, by the method of Burton (Burton, 1956) using calf thymus DNA as a standard. Statistical methods are mentioned with the results.

### Results

The collagen synthesis in intestinal anastomoses is strongly enhanced during the first week after operation. Table I shows both collagen and non-collagen synthesis in ileal anastomoses, as compared to uninjured intestine. The synthetic rate, measured as radioactivity incorporated into the CDP fraction, is significantly increased both 3 and 7 days after

**Table I** Collagen and non-collagen protein synthesis in uninjured and anastomotic ileal tissue

| | *Uninjured intestine* | *3 days after operation* | *7 days after operation* |
|---|---|---|---|
| CDP: | | | |
| d.p.m. $mg^{-1}$wet weight | 70 ± 18 | 509 ± 82[a] | 284 ± 104[a] |
| d.p.m. $\mu g^{-1}$ DNA | 41 ± 7 | 167 ± 64[a] | 184 ± 120[a] |
| NCP | | | |
| d.p.m. $mg^{-1}$wet weight | 4420 ± 820 | 8591 ± 1471[a] | 2841 ± 449 |
| d.p.m. $\mu g^{-1}$ DNA | 2563 ± 343 | 2948 ± 1544 | 1794 ± 778 |
| % RCS | 0.30 ± 0.04 | 1.09 ± 0.21[a] | 1.68 ± 0.67[a] |

Explants from normal and anastomotic ileal tissue were incubated for 3 h with 4.5 μCi $^3$H-proline. Collagen synthesis is expressed as radioactivity in collagenase digestible protein (CDP) and as percentage relative collagen synthesis (RCS). Non-collagen synthesis is expressed as radioactivity in non-collagenous protein (NCP). Data represent average values (± s.d.) from six animals. Differences between anastomoses and control intestine from unoperated rats are tested for significance using a one-sided Wilcoxon test: [a]$0.001 < P \leqslant 0.01$.

**Table II** Collagen and non-collagen protein synthesis in uninjured and anastomotic colonic tissue

| | *Uninjured intestine* | *3 days after operation* | *7 days after operation* |
|---|---|---|---|
| CDP: | | | |
| d.p.m. $mg^{-1}$wet weight | 188 ± 29 | 1002 ± 462[a] | 421 ± 148[a] |
| d.p.m. $\mu g^{-1}$ DNA | 77 ± 8 | 167 ± 88[a] | 154 ± 55[a] |
| NCP | | | |
| d.p.m. $mg^{-1}$wet weight | 6323 ± 1352 | 6637 ± 1371 | 4798 ± 934 |
| d.p.m. $\mu g^{-1}$ DNA | 2622 ± 385 | 1851 ± 943 | 1778 ± 376 |
| % RCS | 0.52 ± 0.03 | 2.65 ± 1.23[a] | 1.48 ± 0.51[a] |

Explants from normal and anastomotic colonic tissue were incubated for 3 h with 4.5 μCi $^3$H-proline. Collagen synthesis is expressed as radioactivity in collagenase digestible protein (CDP) and as percentage relative collagen synthesis (RCS). Non-collagen synthesis is expressed as radioactivity in non-collagenous protein (NCP). Data represent average values (± s.d.) from six animals. Differences between anastomoses and control intestine from unoperated rats are tested for significance using a one-sided Wilcoxon test: [a]$0.001 < P \leqslant 0.01$.

operation. If synthesis is expressed on the basis of wet weight, the increase is 8-fold after 3 days and 4-fold after 1 week. Synthesis of non-collagenous proteins is hardly affected by operation. The only significant difference between anastomoses and uninjured intestine is found after 3 days and when NCP synthesis is calculated on the basis of wet weight. As a consequence, the percentage relative collagen synthesis is also significantly higher in anastomoses than in normal intestine. A similar picture emerges for colonic explants (Table II), although the stimulation of the absolute collagen synthesis appears slightly less pronounced. The increase in CDP, expressed on the basis of tissue weight, is 5- and 2-fold after 3 and 7 days, respectively. Here no significant changes are found in the synthetic rate of non-collagenous proteins.

Administration of cytostatics severely affects postoperative collagen synthesis. Figure 1 depicts the changes in CDP synthesis, expressed on the basis of wet weight. Three days after operation synthesis is significantly lower in all cytostatics groups than in the control group. In ileum, values are suppressed by 43% in the IV group and by 71 and 68% in the IP1 and IP2 group, respectively. Synthetic rates in colonic anastomoses are affected similarly. Seven days after operation, the effect of peri-operative cytostatics persists only in the ileal anastomoses from the animals which had received the drugs intraperitoneally.

The absolute collagen synthesis in the various groups, expressed on the basis of the amount of DNA present, is depicted in Figure 2. Again, incorporation of radioactivity into CDP is strongly inhibited 3 days postoperatively if cytostatics are administered, either intravenously or intraperitoneally, in the peri-operative period. In the IV group the inhibition is no longer apparent after 7 days, while at this time point values in the IP groups remain significantly below those in the control group, both in ileum and in colon.

Figure 3 shows the data for the percentage relative collagen synthesis. Collagen synthesis, as percentage of the total protein synthesis, after 3 days is significantly lower in all cytostatics groups than in the control group. In ileum, the relative collagen synthesis decreases by 32% in the IV group and by 74% in both IP groups. The suppression does not persist at 7 days, with the exception of the ileal anastomoses from the IP2 group. Thus, both absolute and relative anastomotic collagen synthesis are inhibited to approximately the same extent. This can only be the case if the synthesis of non-collagenous proteins remains unaffected by the adminis-

**Figure 2** Effects of cytostatics on collagen synthesis, expressed on a DNA basis, in intestinal anastomosis. Rats were untreated (control group: C) or treated with cytostatics, either intravenously (IV) or intraperitoneally (IP; dose in the IP2 group twice higher than in IV and IP1 groups). Black bars represent values in uninjured intestine from non-operated animals. Average values (±s.d.) from six animals are given. Differences between control and each cytostatics group are tested for significance using a one-sided Wilcoxon test: *$0.01 < P \leqslant 0.05$; **$0.001 < P \leqslant 0.01$; ***$P \leqslant 0.001$.

**Figure 1** Effects of cytostatics on collagen synthesis, expressed per unit wet weight, in intestinal anastomosis. Rats were untreated (control group: C) or treated with cytostatics, either intravenously (IV) or intraperitoneally (IP; dose in the IP2 group twice higher than in IV and IP1 groups). Black bars represent values in uninjured intestine from non-operated animals. Average values (±s.d.) from six animals are given. Differences between control and each cytostatics group are tested for significance using a one-sided Wilcoxon test: *$0.01 < P \leqslant 0.05$; **$0.001 < P \leqslant 0.01$; ***$P \leqslant 0.001$.

**Figure 3** Effects of cytostatics on relative collagen synthesis in intestinal anastomosis. Rats were untreated (control group: C) or treated with cytostatics, either intravenously (IV) or intraperitoneally (IP; dose in the IP2 group twice higher than in IV and IP1 groups). Black bars represent values in uninjured intestine from non-operated animals. Average values (±s.d.) from six animals are given. Differences between control and each cytostatics group are tested for significance using a one-sided Wilcoxon test: *$0.01 < P \leqslant 0.05$; **$0.001 < P \leqslant 0.01$; ***$P \leqslant 0.001$.

**Figure 4** Effects of cytostatics on non-collagen protein synthesis, in 3-days old intestinal anastomosis. Rats were untreated (control group: C) or treated with cytostatics, either intravenously (IV) or intraperitoneally (IP; dose in the IP2 group twice higher than in IV and IP1 groups). Black bars represent values in uninjured intestine from non-operated animals. Average values (±s.d.) from six animals are given.

tration of cytostatics, which fact is illustrated in Figure 4. Indeed, no differences between the control group and the cytostatics groups are observed, with respect to the incorporation of radioactivity into NCP, at 3 days after operation.

It is clear from the preceding data that there exist profound differences between control and cytostatics groups. However, there also exist significant differences between groups depending on the mode of administration of the drugs. Statistical comparison of anastomotic collagen synthesis in the various cytostatics groups shows that the suppressive effects are, almost without exception, greater after intraperitoneal administration than after intravenous administration. This holds for both ileal (Table III) and colonic (Table IV) anastomoses. Although in general both IP groups react similarly, in a few cases differences are also found between groups after low and high doses of intraperitoneal cytostatics. At 7 days after operation, in ileum, suppression of both absolute collagen synthesis, calculated on the basis of DNA, and relative collagen synthesis is significantly more explicit in the IP2 than in the IP1 group. In colon, the only significant difference between both IP groups is for the absolute collagen synthesis, expressed as CDP $\mu g^{-1}$ DNA, after 3 days.

### Discussion

Anastomotic dehiscence is a most serious complication after surgery of the gastrointestinal tract. This phenomenon is still seen rather frequently, even in cases of elective surgery under optimal conditions (Fielding *et al.*, 1980). Since antineoplastic agents interfere with wound healing (Ferguson, 1982), the use of these drugs in the peri-operative period is thought to be a danger to anastomotic integrity. Indeed, our previous experiments confirmed that a 5 day course of 5-fluorouracil, bleomycin and cisplatinum, given intravenously, lowers the mechanical strength of ileal anastomoses constructed on the third day (de Roy van Zuidewijn *et al.*, 1986; 1988). In addition, intraperitoneal administration of the same regimen resulted in impaired strength of both ileal and colonic anastomoses (de Roy van Zuidewijn *et al.*, 1991). The same was found to be true for jejunal anastomoses in rats treated with mitomycin-C (Fumagalli *et al.*, 1991).

**Table III** Significant differences with respect to ileal anastomotic collagen synthesis between the three groups treated with cytostatics

| Collagen synthesis expressed as: | *3 days after operation* | *7 days after operation* |
|---|---|---|
| CDP $mg^{-1}$ wet weight | $IP1<IV^{b}$<br>$IP2<IV^{b}$ | $IP1<IV^{c}$<br>$IP2<IV^{b}$ |
| CDP $\mu g^{-1}$ DNA | $IP1<IV^{c}$<br>$IP2<IV^{b}$ | $IP1<IV^{b}$<br>$IP2<IV^{b}$<br>$IP2<IP1^{a}$ |
| %RCS | $IP1<IV^{c}$<br>$IP2<IV^{c}$ | $IP1<IV^{a}$<br>$IP2<IV^{b}$<br>$IP2<IP1^{a}$ |

Collagen synthesis in ileal anastomotic tissue was measured after incorporation of $^{3}$H-proline. Rats were treated *in vivo* with cytostatics, either intravenously (IV) or intraperitoneally (IP). The concentration of cytostatics in the IV and IP1 group is equal, whereas the concentration in the IP2 group is twice as high. Differences between the groups are tested for significance using a one-sided Wilcoxon test: $^{a}0.01<P\leqslant0.05$; $^{b}0.001<P\leqslant0.01$; $^{c}P\leqslant0.001$.

**Table IV** Significant differences in colonic anastomotic collagen synthesis between the three groups treated with cytostatics

| Collagen synthesis expressed as: | *3 days after operation* | *7 days after operation* |
|---|---|---|
| CDP $mg^{-1}$ wet weight | $IP1<IV^{b}$<br>$IP2<IV^{b}$ | $IP1<IV^{a}$ |
| CDP $\mu g^{-1}$ DNA | $IP1<IV^{b}$<br>$IP2<IV^{a}$<br>$IP1<IP2^{b}$ | $IP1<IV^{a}$<br>$IP2<IV^{a}$ |
| %RCS | $IP1<IV^{b}$<br>$IP2<IV^{b}$ | $IP1<IV^{b}$<br>$IP2<IV^{a}$ |

Collagen synthesis in colonic anastomotic tissue was measured after incorporation of $^{3}$H-proline. Rats were treated *in vivo* with cytostatics, either intravenously (IV) or intraperitoneally (IP). The concentration of cytostatics in the IV and IP1 group is equal, whereas the concentration in the IP2 group is twice as high. Differences between the groups are tested for significance using a one-sided Wilcoxon test: $^{a}0.001<P\leqslant0.01$; $^{b}P\leqslant0.001$.

The bowel wall derives its strength mainly from collagen fibrils located in the submucosa (Thomson *et al.*, 1987; Gabella, 1987). During the first week after surgery anastomotic collagen levels change, supposedly as the result of collagen degradation and synthesis (Hunt *et al.*, 1980). While early anastomotic strength probably depends on the suture holding capacity of the existing collagen fibrils (Högström *et al.*, 1985), newly-formed fibrils should restore pre-operative strength to the sutured bowel. *In vivo* measurements have established the occurrence of increased synthetic activity around experimental anastomoses in ileum (Jönsson *et al.*, 1987) and colon (Jiborn *et al.*, 1980). We have recently confirmed and extended these observations (Martens & Hendriks, 1991), using an *in vitro* technique for measuring collagen synthesis (Martens & Hendriks, 1989) also employed in the present study.

Anastomotic collagen synthesis is strongly enhanced at the time points chosen for this study (Tables I and II). This effect is probably rather specific for collagen since the production of non-collagenous protein hardly changes and, as a result, the percentage relative collagen synthesis increases. We have expressed the results both on the basis of wet weight and on the basis of the amount of DNA present. The incorporation of [$^{3}$H]proline into CDP, expressed per unit DNA, also increases, but to a lesser extent than incorporation expressed per unit weight, in particular 3 days after operation. This indicates that the rise in production of collagen in the wound area is caused not only by an increased number of inflammatory cells, in particular fibroblasts (Hesp *et al.*, 1985), but also by a stimulation of the synthetic rate per cell.

Early anastomotic collagen synthesis is strongly reduced if surgery takes place in the period that the animals receive cytostatics. This phenomenon is observed in both ileum and colon and appears to be specific since the production of non-collagenous protein remains unaffected. Intravenous cytostatics inhibit collagen synthesis to a lesser degree than equal doses given intraperitoneally. The effects also also more transient since 7 days after operation no differences are noted anymore between the control and IV groups, while significant inhibition is still apparent in the IP groups, particularly in ileum. Apparently, intravenous administration results in systemic dilution and thus intraperitoneal administration yields a higher concentration at the anastomotic site. Thus, while intraperitoneal cytostatics may be less harmful in terms of systemic toxicity, this route of administration is more detrimental with respect to collagen synthesis which process is crucial for anastomotic repair. No clinical data are available to support our experimental results. While intravenous administration of 5-fluorouracil alone in the peri-operative periods does not appear to increase the incidence of anastomotic leakage (Taylor *et al.*, 1985; Klausner *et al.*, 1986), no results are known as yet for intraperitoneal chemotherapy. Our present data, together with those published before (de Roy van Zuidewijn *et al.*, 1991) indicate that the latter mode of administration would carry greater risks for anastomotic integrity.

On the whole, we found few differences between the IP1 and IP2 groups. Doubling the dose does not result in further inhibition of either absolute or relative collagen synthesis as measured 3 days after operation. Presumably, there exists a basal level of synthesis, almost equal to that observed in the uninjured intestine, which remains unaffected by chemotherapy. One effect of the higher dose is that the inhibitory effect is prolonged: a significantly stronger inhibition is noted in 7 days old ileal anastomoses from the IP2 group. This could be explained by more sustained inhibitory concentrations, although it remains puzzling why this does not affect the colonic anastomoses.

In earlier studies we found that peri-operative administration of cytostatics induced a decreased anastomotic collagen content (de Roy van Zuidewijn *et al.*, 1986; 1988; 1991). Although we have not measured collagen degradation under these conditions, the present results strongly suggest that they are caused by a severe inhibition of the synthesis. In one of these studies (de Roy van Zuidewijn *et al.*, 1991) we reported that different doses of intraperitoneal cytostatics, equal to those used in the present study, had divergent effects on the mechancial strength of intestinal anastomoses: the higher dose induced significantly more loss of strength. No such clear differences are found between the IP1 and IP2 groups with respect to collagen synthesis. This fact supports the hypothesis (Hendriks & Mastboom, 1990) that the amount of collagen present is not the only, and perhaps not the most decisive, factor deciding anastomotic strength. Certainly the quality of the collagen, in particular its intra- and intermolecular crosslinks, are important. It could very well be that increasing doses of cytostatics interfere with crosslinking without affecting the rate of synthesis of collagen monomers.

Although it has been shown that endothelial cells from the rat small intestine (Quaroni & Trelstad, 1980) and smooth muscle cells from the human jejunum are capable of synthesising collagen (Graham *et al.*, 1987), it seems likely that the fibroblasts are primarily responsible for the increased collagen production in the wound area. The number of fibroblasts is strongly increased from 2 days after operation onwards (Hesp *et al.*, 1985). The mechanisms which are responsible for the suppression of collagen synthesis are as yet unknown. Fibroblast chemotaxis could be affected, either directly or through inhibition of macrophage function. We have found histological evidence that the number of fibroblasts in ileal anastomoses is decreased by cytostatics (de Roy van Zuidewijn *et al.*, submitted). However, cytostatics could also interfere directly with fibroblast protein synthesis. It has been shown that doxorubicin inhibits collagen synthesis in fibroblast cultures (Sasaki *et al.*, 1987).

Thus, this study indicates that the cytostatic regimen used severely inhibits anastomotic collagen synthesis, which effect probably causes the delay in the development of mechanical strength, reported before. The effects of intravenous administration are less severe and less persistent than those of equivalent doses given intraperitoneally. We believe that the present data support the notion that peri-operative intraperitoneal cytostatics are harmful to anastomotic repair in the intestine.

The authors are grateful to B.M. de Man for expert technical assistant and to Th.M. de Boo for statistical analysis of the data.

### References

BURTON, K. (1956). A study of the conditions and mechanisms of the diphenylamine reaction for the colorimetric estimation of deoxy-ribonucleic acid. *Biochem. J.*, **62,** 15.

CUNLIFFE, W.J. & SUGARBAKER, P.H. (1989). Gastrointestinal malignancy rationale for adjuvant therapy using early postoperative intraperitoneal chemotherapy. *Br. J. Surg.*, **76,** 1082.

DEDRICK, R.L., MYERS, C.E., BUNGAY, P.M. & DEVITA, V.T. (1978). Pharmacokinetic rationale for peritoneal drug administration in the treatment of ovarium cancer. *Cancer Treat. Rep.*, **62,** 1.

FERGUSON, M. (1982). The effect of antineoplastic agents on wound healing. *Surg. Gynecol. Obstet.*, **154,** 421.

FIELDING, F.P., STEWART-BROWN, S., BLESOVSKY, L. & KEARNEY, G. (1980). Anastomotic integrity after operations for large bowel cancer: a multicentre study. *Br. Med. J.*, **281,** 411.

FUMAGALLI, U., TRABUCCHI, E., SOLIGO, M. & 4 others (1991). Effects of intraperitoneal chemotherapy on anastomotic healing in the rat. *J. Surg. Res.*, **50,** 82.

GABELLA, G. (1987). The cross-ply arrangement of the collagen fibers in the submucosa of the mammalian small intestine. *Cell Tissue Res.*, **248,** 491.

GOLDIE, J.H. & COLDMAN, A.J. (1979). A mathematic model for relating the drug sensitivity of tumors to their spontaneous mutation rate. *Cancer Treat. Rep.*, **63,** 1727.

GOLDIE, J.H. & COLDMAN, A.J. (1984). The genetic origin of drug resistance in neoplasms: implications for systemic therapy. *Cancer Res.*, **44,** 3643.

GRAHAM, M.F., DRUCKER, D., DIEGELMANN, R.F. & ELSON, C. (1987). Collagen synthesis by human intestinal smooth muscle cells in culture. *Gastroenterology*, **92,** 400.

GRAHAM, M.F., DIEGELMANN, R.F., ELSON, C.O. & 4 others (1988). Collagen content and types in the intestinal strictures of Crohn's disease. *Gastroenterology*, **94,** 257.

GUNDUZ, N., FISHER, B.V. & SAFFER, E.A. (1979). Effect of surgical removal on the growth and kinetics of residual tumor. *Cancer Res.*, **39,** 3861.

HENDRIKS, Th. & MASTBOOM, W.J.B. (1990). Healing of experimental intestinal anastomoses: parameters of repair. *Dis. Colon Rectum*, **30,** 891.

HESP, W.L.E.M., HENDRIKS, Th., SCHILLINGS, P.H.M., LUBBERS, E.J.C. & DE BOER, H.H.M. (1985). Histological features of wound repair: a comparison between experimental ileal and colonic anastomoses. *Br. J. Exp. Pathol.*, **66,** 511.

HÖGSTROM, H., HAGLUND, U. & ZEDERFELDT, B. (1985). Suture technique and early breaking strength of intestinal anastomoses and laparotomy wounds. *Acta Chir. Scand.*, **151,** 441.

HUNT, T.K., HAWLEY, P.R., HALE, J., GOODSON, W. & THAKRAL, K.K. (1980). Colon repair: the collagenous equilibrium. In *Wound Healing and Wound Infection.* Theory and Surgical Practice. Hunt, T.K. (ed.), p. 153. Appleton-Century-Croft, New York.

JIBORN, H., AHONEN, J. & ZEDERFELDT, B. (1980). Healing of experimental colonic anastomoses. IV. Effect of suture technique on collagen metabolism in the colonic wall. *Am. J. Surg.*, **139,** 398.

JÖNSSEN, K., JIBORN, H. & ZEDERFELDT, B. (1987). Collagen metabolism in small intestinal anastomoses. *Am. J. Surg.*, **154,** 288.

KLAUSER, J.M., LELCUH, S., INBAR, M. & ROZIN, R. (1986). The effects of perioperative fluorouracil administration on convalescence and wound healing. *Arch. Surg.*, **121,** 239.

MARTENS, M.F.W.C. & HENDRIKS, Th. (1989). Collagen synthesis in explants from rat intestine. *Biochim. Biophys. Acta.*, **993,** 252.

MARTENS, M.F.W.C. & HENDRIKS, Th. (1991). Postoperative changes in collagen synthesis in intestinal anastomoses of the rat: differences between small and large bowel. *Gut*, **32,** 1482.

MARTIN, D.S. (1981). The scientific basis for adjuvant chemotherapy. *Cancer Treat. Rev.*, **8,** 169.

MULDER, J.H., DE RUITER, J. & EDELSTEIN, M.B. (1983). Model studies in adjuvant chemotherapy. *Cancer Treat. Rep.*, **67,** 45.

PETERKOFSKY, B., CHOJKIER, M. & BATEMAN, J. (1981). Determination of collagen synthesis in tissue and cell culture systems. In *Immunochemistry of the Extracellular Matrix.* Volume 2. Furthmayer, H. (ed.), p. 19. Boca Raton: CRC Press.

QUARONI, A. & TRELSTAD, R.L. (1980). Biochemical characterization of collagens synthesised by intestinal epithelial cell culture. *J. Biol. Chem.*, **17,** 8351.

DE ROY VAN ZUIDEWIJN, D.B.W., WOBBES, Th., HENDRIKS, Th., KLOMPMAKERS, A.A. & DE BOER, H.H.M. (1986). The effect of antineoplastic agents on the healing of small intestinal anastomoses in the rat. *Cancer*, **58**, 2.

DE ROY VAN ZUIDEWIJN, D.B.W., WOBBES, Th., HENDRIKS, Th., KLOMPMAKERS, A.A. & DE BOER, H.H.M. (1988). *Surg. Res. Comm.*, **2**, 297.

DE ROY VAN ZUIDEWIJN, D.B.W., HENDRIKS, Th., WOBBES, Th. & DE BOER, H.H.M. (1991). Effects of intraperitoneal cytostatics on the healing of experimental intestinal anastomoses. *Br. J. Cancer*, **63**, 937.

SASAKI, T., HOLEYFIELD, K.C. & UITTO, J. (1987). Doxorubicin induced inhibition of prolyl hydroxylation during collagen biosynthesis in human skin fibroblasts cultures. *J. Clin. Invest.*, **80**, 1735.

TAYLOR, I., MACHIN, D., MULLEE, M., TROTTER, G., COOKE, T. & WEST, C. (1985). A randomized controlled trial of adjuvant portal vein cytotoxic perfusion in colorectal cancer. *Br. J. Surg.*, **72**, 359.

THOMSON, H.J., BUSUTTIL, A., EASTWOOD, A.N. & ELTON, R.A. (1987). Submucosal collagen changes in the normal colon and in diverticular disease. *Int. J. Colorectal Dis.*, **2**, 208.